# Henry Rose Carter

**DOI:** 10.3201/eid1510.090129

**Published:** 2009-10

**Authors:** Myron G. Schultz

**Affiliations:** Centers for Disease Control and Prevention, Atlanta, Georgia, USA

This is a photograph of Henry Rose Carter (1852–1925). It shows him in his Public Health Service uniform sometime after 1915, when he attained the rank of Assistant Surgeon General. Carter was a quarantine officer and a field epidemiologist, best known for his discovery in 1898 of the extrinsic incubation period of yellow fever. His discovery led directly to the historic finding by Reed, Carroll, Agramonte, and Lazear in 1900 that mosquitoes are the intermediary hosts of the infecting agent that causes yellow fever.

A member of a distinguished Virginia family, Henry Rose Carter was born in Caroline County, Virginia, on August 25, 1852. He graduated as a civil engineer at the University of Virginia; took special courses in mathematics and applied chemistry at the same institution; and studied medicine at the University of Maryland, graduating in 1879. That same year, Carter joined the Marine Hospital Service (MHS), later the United States Public Health Service. Carter's initial MHS assignments placed him at the center of the yellow fever maelstrom. In 1879, he was detailed to Memphis and other southern cities, then in the throes of a second year of devastating epidemics. There began his lifelong interest in the epidemiology and control of yellow fever.

The Deep South of the United States, where Carter conducted most of his work on yellow fever, was affected by periodic outbreaks of the disease. This highly fatal disease had long been a scourge in the United States, especially in port cities. Lack of scientific understanding fueled these outbreaks. In 1793, for example, when a major outbreak of yellow fever struck Philadelphia, Benjamin Rush, the leading resident doctor, thought the fever came from a batch of spoiled coffee from a ship in the harbor. Not until 1900 did Walter Reed and his colleagues solve the mystery by proving that yellow fever was spread solely by mosquitoes. Until then only a few people, including Carter, were pursuing the cause of yellow fever in a methodical, scientific way.

In 1888, Carter was assigned to the Gulf Coast Maritime Quarantine Station at Ship Island, Mississippi. Here, and at subsequent quarantine station postings along the US Gulf Coast, he thoroughly reviewed the rationale for quarantine policies with a view toward establishing uniform regulations and more thorough disinfection of vessels and minimizing interference with naval commerce. Crucial to the success of these activities was Carter's attention to the incubation period of yellow fever. Carter ably directed the MHS epidemiologic control efforts in numerous threatened regions throughout the South. Contemporaries described Carter as a man of great energy who would work in swamps 10–15 hours a day, often forgetting to shed his clothes when he went to sleep at night. He was completely absorbed in the study of mosquitoes and, from the standpoint of control and sanitation, probably knew more about them than anyone else at that time. He resembled his contemporary, Teddy Roosevelt. Like Roosevelt, he was of less than medium height and solid of physique, wore eyeglasses over a strong nose, and sported a handlebar moustache in a red face.

In conjunction with the sanitary work for the 1898 season, Carter made detailed notes on the development of yellow fever in the rural communities of Orwood and Taylor, Mississippi. The circumstances under which Carter worked were favorable for recording the time between the onset of infection among persons in isolated farmhouses and the occurrence of secondary cases among others in these same houses. Carter observed that when a case of yellow fever occurred in an isolated farmhouse, persons who visited the house at the time did not acquire the disease, but those who arrived 2 weeks later were susceptible to infection. Wade Hampton Frost, a fellow commissioned officer and later a distinguished professor of epidemiology, wrote that one needs to study all of Carter’s papers to appreciate his patient persistence in collecting material, his scrupulous care in excluding every observation that might be subject to any question, his skill in analyzing the complex and puzzling data, and his clear logic in establishing a conclusion so remarkable as the existence of a definite period of “incubation in the environment.” According to Carter:

The period from the first (infecting) case to the first group of cases infected at these houses is generally from two to three weeks. The houses having become infected, susceptible individuals who visited them for a few hours fell sick with the disease in the usual period of incubation—one to seven days.

These observations pointed to the presence of an intermediate host, such as the mosquito, which having taken an infecting agent into its stomach soon after entrance of the patient into a noninfected house, was able after a certain interval to retransmit the infecting agent to other persons. Carter called this interval between the primary and secondary cases “the period of extrinsic incubation,” and defined its usual range as 10–17 days.

In 1899, before he was able to publish his conclusions, Carter was assigned to Cuba. He served there as the Chief Quarantine Officer for the MHS in the aftermath of the Spanish–American War. This assignment was fortuitous because there he met Jesse Lazear, 1 of the 4 members of the US Army Yellow Fever Commission headed by Walter Reed. Carter had finally arranged for his paper's publication that year in the New Orleans Medical and Surgical Journal and gave a draft to Lazear. “If these dates are correct,” Carter later recalled Lazear saying, “it spells a living host.” The theory that mosquitoes are the vectors of yellow fever was first advanced in the United States by Dr J.C. Nott of Mobile, Alabama, who in 1848 wrote a paper titled “On the Cause of Yellow Fever” in which he stated his belief that insects play a role as carriers of yellow fever. In the late 19th century, the renowned Cuban physician and scientist Carlos J. Finlay devoted 2 decades to attempting to prove that mosquitoes are the vectors of yellow fever. Periodic epidemics of yellow fever ravaged the population of Finlay’s native Cuba, particularly affecting the citizens of Havana, where he had set up a medical practice in 1864. During 1881–1900, Finlay carried out 102 experimental inoculations of human volunteers to prove his hypothesis. He believed he had produced some cases of yellow fever by mosquito inoculation, but the public health community remained skeptical.

One criticism of Finlay’s work was that participants were never sufficiently isolated from the general population to eliminate the possibility of contracting yellow fever from sources other than Finlay’s experimental mosquitoes. This, and the inconsistency with which fevers developed in the volunteers, kept the mosquito theory on the margins of acceptability. Most important, Finlay’s experiments missed 2 essential parts of the development of the agent of yellow fever in mosquitoes: Finlay did not consistently apply mosquitoes to yellow fever patients during the first 3 days of their illnesses, i.e., the period of viremia, and he applied mosquitoes by feeding them on his volunteers too soon after they were presumably infected by feeding on yellow fever patients. He never considered the possibility of an extrinsic incubation period that would require time for the agent of yellow fever to incubate within mosquitoes.

The importance of Carter’s observations in determining the direction of Reed’s experimental work is contained in Reed, Carroll, Agramonte, and Lazear’s first publication on the etiology of yellow fever in October 1900. The authors stated that 3 considerations made them turn their attention to the theory of transmission by mosquitoes:

1. Certain general facts in the epidemiology of the disease (chiefly its sharp seasonal and geographic limitations) that had led Finlay to formulate his theory of mosquito transmission.
2. The work of Ross and the Italian researchers Grassi, Bastianelli, and Bignami in demonstrating the conveyance of malaria by the mosquito (development of parasite in the mosquito; limitation to 1 genus of mosquitoes).
3. Carter’s observation (1898) on the extrinsic incubation period.

Reed and his colleagues had the good fortune to begin their investigations with a correct hypothesis that was based on the three considerations cited above. By 1900, the Reed Commission had established the following facts relating to yellow fever: 1) yellow fever is transmitted by a mosquito, now known as *Aedes aegypti*; 2) to become infected, the mosquito must feed on the yellow fever patient during the first 3 days of the disease; 3) the mosquito does not become infective until 10–16 days after it takes blood from a yellow fever patient; and 4) the incubation period in humans, i.e., the time between the moment a person is bitten by an infective mosquito and the time a person’s symptoms appear, does not exceed 6 days.

The third point had been established chiefly through the investigations by Carter. He called this 10–16-day interval that occurs in the mosquito the “period of extrinsic incubation.” Carter’s discovery of the extrinsic incubation period of yellow fever places him with Reed and Gorgas in the distinguished group of scientists and sanitarians who have made the most significant contributions to our knowledge of this disease and the methods of combating it. Walter Reed saluted Carter when he said, “I know of no one more competent to pass judgment on all that pertains to the subject of yellow fever. You must not forget that your own work in Mississippi did more to impress me with the importance of an intermediate host in yellow fever than everything else put together.”
